# Developmental plasticity of texture discrimination following early vision loss in the marsupial *Monodelphis domestica*

**DOI:** 10.1242/jeb.236646

**Published:** 2021-05-13

**Authors:** Deepa L. Ramamurthy, Heather K. Dodson, Leah A. Krubitzer

**Affiliations:** 1 Center for Neuroscience, University of California, Davis, Davis, CA 95618, USA; 2 Department of Psychology, University of California, Davis, Davis, CA 95618, USA

**Keywords:** Active touch sensing, Compensatory plasticity, Short-tailed opossum, Texture, Vibrissae

## Abstract

Behavioral strategies that depend on sensory information are not immutable; rather they can be shaped by the specific sensory context in which animals develop. This behavioral plasticity depends on the remarkable capacity of the brain to reorganize in response to alterations in the sensory environment, particularly when changes in sensory input occur at an early age. To study this phenomenon, we utilize the short-tailed opossum, a marsupial that has been a valuable animal model to study developmental plasticity due to the extremely immature state of its nervous system at birth. Previous studies in opossums have demonstrated that removal of retinal inputs early in development results in profound alterations to cortical connectivity and functional organization of visual and somatosensory cortex; however, behavioral consequences of this plasticity are not well understood. We trained early blind and sighted control opossums to perform a two-alternative forced choice texture discrimination task. Whisker trimming caused an acute deficit in discrimination accuracy for both groups, indicating the use of a primarily whisker-based strategy to guide choices based on tactile cues. Mystacial whiskers were important for performance in both groups; however, genal whiskers only contributed to behavioral performance in early blind animals. Early blind opossums significantly outperformed their sighted counterparts in discrimination accuracy, with discrimination thresholds that were lower by ∼75 μm. Our results support behavioral compensation following early blindness using tactile inputs, especially the whisker system.

## Abstract (precis)

**Summary:** Whisker-mediated touch guides texture discrimination in short-tailed opossums. Texture discrimination sensitivity and associated tactile strategies in these marsupials show behavioral plasticity, which is dependent on sensory inputs available during development.

## INTRODUCTION

The behavioral strategies that animals use to orient themselves and navigate within complex environments are highly dependent on the sensory context in which they live. Over the long time course of evolution, different species exhibit sensory adaptations to the ecological niche they occupy. For example, many subterranean mole rats that dwell in burrow systems, with little to no light, are naturally blind, with microphthalmic eyes and no functional vision (Cooper et al., 1993). Instead, they rely heavily on touch and audition, accompanied by a corresponding magnification of sensory representations associated with these senses in cortical and subcortical structures (Mann et al., 1997; Bronchti et al., 2002; Kimchi and Terkel, 2004; Sadka and Wollberg, 2004; Kimchi et al., 2005; Park et al., 2007). In fact, what would normally be visual cortex is co-opted by somatosensory and auditory systems. While evolutionary history places some constraints on the sensory-mediated behaviors that animals can engage in, over shorter time scales behaviors can still be strongly influenced by the sensory context in which an animal develops (Montero, 1997; Arkley et al., 2014). Humans are naturally visual animals (Preuss, 2003); however, individuals who experience vision loss, especially in cases of congenital deficits, can become highly effective at using tactile and auditory based strategies along with perceptual learning that manifest as heightened sensitivity to stimuli mediated by the spared senses (Goldreich and Kanics, 2003; Voss, 2011; Wong et al., 2011; for review, see Bavelier and Neville, 2002; Merabet and Pascual-Leone, 2010; Kupers and Ptito, 2011; Renier et al., 2014; Ricciardi et al., 2014). Finally, even without the loss of a sensory receptor array, any given individual might rely more heavily on one sensory strategy over another in different settings, depending on the ongoing availability and behavioral relevance of sensory information (Montero, 1997; Lee et al., 2016); for example, touch and hearing may be prioritized over vision upon entering a dark room or at night, when visual information is not readily available.

To appreciate the extent to which early sensory context can shape tactile-mediated behavior, we experimentally altered the relative weights of different sensory inputs in short-tailed opossums through bilateral enucleation at a very early developmental stage (postnatal day 4; P4). Enucleation at P4 in opossums is prior to the onset of spontaneous activity in the retina, and before the formation of retinofugal and thalamocortical pathways (Taylor and Guillery, 1994; Molnar et al., 1998). Previous studies in our laboratory have shown that this manipulation results in a major functional reorganization of visual cortex and alterations in its cortical and subcortical connections (Kahn and Krubitzer, 2002; Karlen et al., 2006; Karlen and Krubitzer, 2009), as well as anatomical and physiological alterations in somatosensory cortex (Kahn and Krubitzer, 2002; Karlen et al., 2006; Karlen and Krubitzer, 2009; Ramamurthy and Krubitzer, 2018; Dooley and Krubitzer, 2019). These changes bear a strong resemblance to cortical organization observed in naturally blind animals (Halley and Krubitzer, 2019). Notably, neurons in primary somatosensory cortex (S1) of early blind opossums are more selective in their responses to single whisker stimuli and exhibit improved discriminability of whisker identity (Ramamurthy and Krubitzer, 2018). This could support enhanced discrimination of tactile features on a spatial scale, that is at or above the distance between neighboring whiskers (Diamond, 2010). However, it is unknown if early blind opossums are better at sensing and discriminating textures on a very fine spatial scale, and critically, whether this manifests at the behavioral level.

In their natural habitat, short-tailed opossums are semi-arboreal and crepuscular (Eisenberg and Redford, 1989; Jones et al., 2003; Macrini, 2004; Carvalho et al., 2011), preferring low light conditions (Seelke et al., 2014). Under such circumstances, texture becomes especially crucial as a sensory cue, given the paucity of visual cues in dim light. Short-tailed opossums use texture cues to adjust their locomotor kinetics and footfall patterns as behavioral strategies to maintain balance on arboreal substrates (Lammers, 2004, 2007, 2009; Lammers and Biknevicius, 2004; Lammers et al., 2006; Lammers and Gauntner, 2008). In most small mammals, the facial whiskers are a major channel for gathering sensory information about position, shape and texture of objects in the immediate vicinity of the animal, and are essential for navigation in complicated and irregular settings – especially in the dark (Vincent, 1912; Pocock, 1914; Huber, 1930a,b; Lyne, 1959; Russell and Pearce, 1971; Ahl, 1986; Diamond et al., 2008; Diamond and Arabzadeh, 2013; Sarko et al., 2013; Englund et al., 2020). When opossums are deprived of visual input at an early age, they are forced to become even more heavily reliant on whisker input as the primary means of exploring and navigating their environment (Ramamurthy and Krubitzer, 2018; Englund et al., 2020). Compensation for blindness through whisker-mediated touch has also been studied in rodent models. Neonatally enucleated rats were faster at finding their way through a maze, relative to sighted rats (Toldi et al., 1994). Functionally blind rats (dystrophic RCS rats; Arkley et al., 2014) and mice (5xFAD mutant mice; Grant et al., 2020) with early onset retinal degeneration showed different whisker kinematic strategies relative to sighted controls during exploration of novel environments, and when challenged by obstacles in those environments.

Short-tailed opossums have two prominent sets of facial whiskers – the mystacial whiskers (located on the snout) and the genal whiskers (located on the cheek), both of which are involved in tactile exploration through active whisking (Grant et al., 2013; Ramamurthy and Krubitzer, 2016). As in rodents, their mystacial whisker pads have specialized musculature for protraction and retraction, allowing high degrees of freedom in the control of whisker position during tactile behavior (Mitchinson et al., 2011; Grant et al., 2013). Studies on rodents have found that the mystacial whiskers can be used for texture discrimination in both active and passive touch (Park et al., 2020). Genal whiskers in opossums exhibit whisking movements that are in general synchrony with mystacial whisker motion, but they have their own musculature (Grant et al., 2013) and can move independently of the mystacial set. The use of genal whiskers in texture discrimination has not been previously studied.

In the current study, we examined the role of the mystacial whiskers and genal whiskers in guiding tactile-based decisions in early blind and sighted opossums, and measured their behavioral discrimination sensitivity to texture cues. To our knowledge, this is the first psychometric investigation of texture discrimination in a marsupial. Moreover, we compare results from sighted animals with those from bilaterally enucleated opossums performing the same task to examine the extent to which developmental history has an impact on texture discrimination sensitivity and the role of facial whiskers in guiding behavior.

## MATERIALS AND METHODS

### Animals

Behavioral experiments were performed in six adult short-tailed opossums [*Monodelphis domestica* (Wagner 1842); three males, three females; 4–16 months] obtained from two separate litters. Three of the six animals were bilaterally enucleated at P4 (see below) and were part of a larger study on developmental plasticity of sensory cortex. See Table 1 for details regarding full sample numbers per individual animal for each testing condition. All experimental procedures were approved by the UC Davis Institutional Animal Care and Use Committee and conform to NIH guidelines.

**Table 1.**
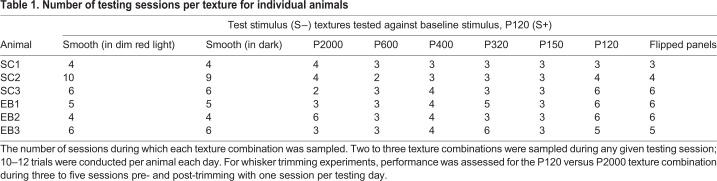
Number of testing sessions per texture for individual animals

### Bilateral enucleations

Bilateral enucleations were performed at P4 using procedures that have been previously described (Ramamurthy and Krubitzer, 2018). Mothers of experimental litters were lightly anesthetized with Alfaxan (initial dose: 20 mg kg^−1^; maintenance doses: 10–50% i.m.) to facilitate enucleation of the pups, which are attached to the mother's nipples at this developmental stage. Pups were anesthetized by hypothermia. Health of both the mother (respiration rate and body temperature) and the pups (heartbeat, coloration and mobility) was monitored throughout the procedure. An incision was made in the skin covering the eyes, the eyes were removed under microscopic guidance, and the skin was repositioned and resealed using surgical glue. Approximately 50% of each litter was bilaterally enucleated. After complete recovery from anesthesia, mothers along with their attached litters were returned to their home cages.

### Testing apparatus and procedure

**Fig. 1.**
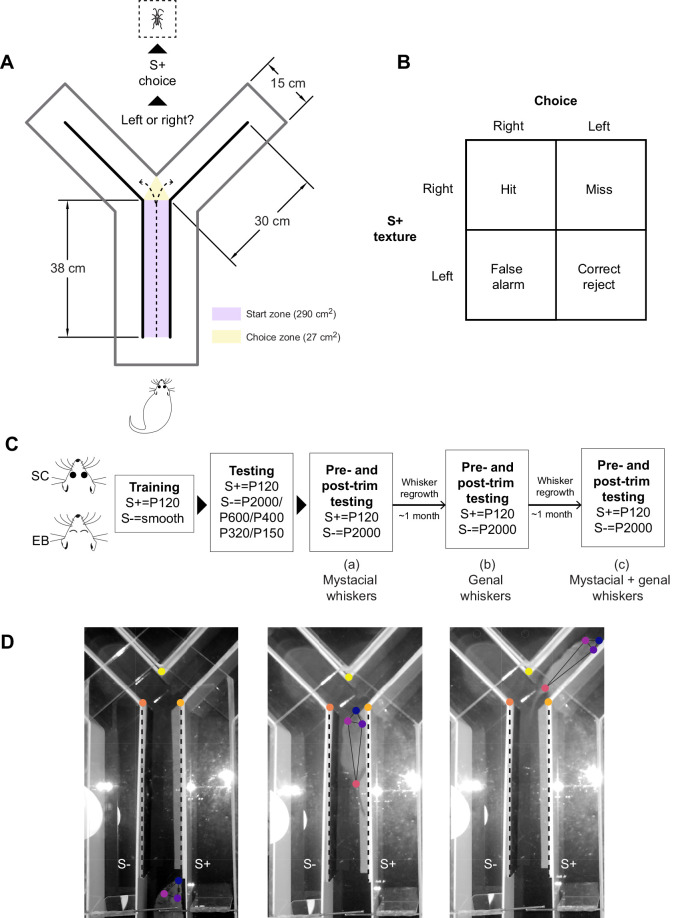
**Experimental design.** (A) Behavioral testing apparatus. A Y-maze was modified to test texture discrimination capabilities using a two-alternative forced choice paradigm (2AFC). Stimulus panels were attached to the inner walls of the Y-maze (thick black lines). Opossums were required to discriminate between a baseline sandpaper (120 grit sandpaper, S+) and a range of sandpapers of different grits (test stimuli, S−). Choice of the S+ stimulus was rewarded with a live cricket. The start zone and choice zone were designated as shown for the quantification of texture discrimination latency. (B) Contingency table showing trial types, response types and trial outcomes that result from all possible combinations of trial type and response type. On any given trial, the S+ texture can be presented on the left or right side of the maze (left/right trial types) and the animal can respond by choosing either the left or the right arm (left/right choice) in the 2AFC texture discrimination task paradigm. When S+ was presented on the right, choice of the right arm was counted as a hit, while choice of the left arm was a miss. When S+ was presented on the left, choice of the right arm counted as a false alarm, while choice of the left arm was a correct rejection. (C) Training and testing timeline. Following initial training and testing across all stimulus combinations, the contribution of the facial whiskers to performing this task was determined by trimming (a) mystacial whiskers, (b) genal whiskers and (c) mystacial and genal whiskers. (D) Video stills showing the progression of an opossum through the Y-maze on a single trial. Markers used for tracking positional landmarks, namely the vertices of choice zone (yellow hues), as well as markers used to track the opossum's body (blue and purple hues, head and tail base), are shown. The simple posture skeleton built from using these markers is overlaid (black continuous lines). The position of stimulus panels in the start zone is also indicated (black dashed lines).

Animals were tested in a Y-maze modified to test texture discrimination capabilities using a two-alternative forced choice paradigm (2AFC) (Fig. 1A–D). A similar approach has previously been used in laboratory rodents (Smith, 1939; Finger and Frommer, 1968; Finger et al., 1970; Lipp and Van der Loos, 1991; Hughes, 2007). The Y-maze (Fig. 1A) was custom-built from plexiglass, and consisted of a corridor [38 cm (15 inches) long, 30 cm (12 inches) high] and two arms [30 cm (12 inches) long, 30 cm (12 inches) high]. A clear plexiglass lid covered the top of the maze. The animal was required to discriminate between a baseline sandpaper (120 grit sandpaper, S+) and a range of sandpapers of different grits (test stimuli, S−). Both the baseline and test stimuli consisted of removable textured panels that could be attached to either arm of the Y-maze, and their respective sides of the corridor. Panels were made of polystyrene and covered with sandpaper of different grit sizes (corresponding to S− stimuli or S+ stimulus). The ‘goal arm’ was defined as the arm which contained the S+ stimulus. For each trial, entry into the ‘goal arm’ was considered a correct choice (hit), while entry into the non-goal arm was considered an incorrect choice (miss). After completion of each trial, the opossum returned to the start zone to begin a new trial. A hit was rewarded with a live cricket. The order of presentation of the different textures was pseudorandomized and 10–12 trials were conducted per animal each day. The maze was cleaned with ethanol in between trials to eliminate olfactory cues. If an animal did not consume the reward within 1 min following successful trial completion on >50% of hit trials, data from that day were excluded due to low reward-driven motivation for accurately performing the task. All testing was conducted either in dim red light (660 nm: outside the expected peak sensitivity range of cone visual pigments in short-tailed opossums; Hunt et al., 2009) or in the dark, guided by a night vision camera. Exposure to the two light conditions was balanced such that roughly half of the total sessions per group fell within each condition [percentage of total sessions under dim red light: sighted controls (SC), 51.3%; early blind (EB), 50%]. In a subset of trials, video recordings were captured under infrared lighting using a night vision camera (Seree Night Vision HDV-501 1080P, 60 fps). These were used for analysis of maze exploration and decision latency in opossums.

### Training stages

Behavior was shaped gradually over a series of training stages described below in sequence.

#### Handling

Handling began immediately following weaning (P56) to facilitate training and testing on the texture discrimination task in adulthood. Handling occurred 2–3 days per week, from weaning until adulthood (for developmental timeline, see Ramamurthy and Krubitzer, 2018). All animals were handled for equal amounts of time (2 min) per day.

#### Acclimation to the testing room

Animals were transported to the testing room in their home cages and allowed to acclimate there for around 10 min. This phase lasted for 1 day.

#### Acclimation to the testing apparatus

Animals were placed in an open arena in the testing room and then repeatedly moved to and from the testing apparatus (Y-maze) for a total of 2 min per day. This process was repeated 3–5 times a week for 30 days.

#### Stimulus–reward pairing

Animals were placed in the open arena, now half covered by the roughest sandpaper (P120 grit). Approach towards and contact with the sandpaper was positively reinforced using a food reward (live cricket), paired with a sound (click). This stage of training lasted for ∼2 weeks.

#### Two-alternative forced choice training

Animals were placed in the start zone (Fig. 1A) and allowed to navigate the Y-maze. The roughest sandpaper (P120 grit; mean grain size of 125 µm; ISO 6344 industrial standard) was used in the goal arm during this phase. Choice to enter the goal arm was positively reinforced by a click sound, followed by delivery of a cricket as the food reward. This phase lasted until criterion (70% accuracy) was achieved.

#### Two-alternative forced choice testing

The testing phase began once animals reached criterion performance (70% accuracy). In this phase, the smooth panels were replaced by panels covered with sandpaper of varying roughness (P2000, P600, P400, P320 and P150, with mean grain sizes of 10.3, 25.8, 35, 46.2 and 100 µm, respectively; ISO 6344 industrial standard). The contrast in textures between the goal arm (P120 grit) and the non-goal arm (P120–P2000) could thus be varied across trials to measure psychometric functions of texture discrimination. Furthermore, data were also acquired on days when either the same texture was presented on both sides, or a P120 versus P2000 combination was presented with the panels flipped. This allowed us to control for the possibility of choices being guided by olfactory cues from the sandpaper (Finger et al., 1970). In the flipped panel configuration, the stimulus panels were affixed such that the sandpaper-covered side faced the wall of the Y-maze and the smooth backing of the panel was instead facing inwards, towards the animal. Thus texture-related cues associated with the stimulus panels were obscured while any odorant cues associated with the sandpaper would be retained. For trials in which either P120 texture was presented in both arms or when flipped panels were used to control for olfactory cues, either the right or the left panel was randomly selected to be the S+ stimulus prior to the start of the session and rewarded accordingly.

### Whisker trimming experiments

Animals were not trained to use any specific body part to perform the texture discrimination. However, the height of the maze was such that it prevented animals from rearing up and actively palpating the stimulus panels with their forepaws. Like mice and rats, short-tailed opossums naturally whisk during locomotion and object exploration. The goal of these experiments was to test the extent to which these animals naturally favored the use of their whiskers for texture discrimination to guide behavior.

Following data collection across all tested stimulus combinations, we determined the contribution of the mystacial whiskers and the genal whiskers in performing this task by trimming each set of whiskers separately or at the same time (see Fig. 1C for training and testing timeline). Three sets of whisker trimming experiments were performed: (1) mystacial whiskers only, (2) genal whiskers only, and (3) both mystacial and genal whiskers. Whiskers were bilaterally trimmed down to approximately 0.5 mm in length (under light isoflurane anesthesia; 1–2% for 3–5 min), and the acute effect on behavior was measured for 3–4 days following whisker trimming. Each set of whisker trimming experiments was separated by at least 1 month to allow complete regrowth of all whiskers between experiments. To maintain the stimulus–reward association, some training sessions occurred at <1 month, but these data were not included in the analysis as whisker regrowth could be partial in those cases. Three to five sessions of data on texture discrimination performance on P120 versus P2000 combination was acquired pre-trimming, followed by three to five sessions for the same stimuli post-trimming. Sham procedures mimicking whisker trimming (brushing rather than trimming whiskers under 1–2% isoflurane for 3–5 min) were included prior to the pre-trimming phase. The first post-trimming behavioral session was conducted no sooner than 18 h after the whiskers were trimmed.

### Video analysis

#### Manual scoring

All video data were manually scored by two independent observers using BORIS (Behavioral Observation Research Interactive) software (version 4.1.4). The parameters scored were: (1) total time spent in the start zone and (2) total time spent in the choice zone until the animal left the choice zone and entered one of the arms. The time point of entry into a zone was recorded as the time at which the tip of the snout first entered the zone, while the time point of exit (Fig. 1A) was recorded as the time at which the whole body of the animal (except the tail) left that zone. For whisker trimming experiments, video data were compared between the last pre-trim session and first post-trim session for all animals in each experimental group during mystacial and mystacial+genal trimming experiments, and for a subset of animals (2 EB, 1 SC) during the genal whisker trimming experiment due to video acquisition issues.

#### Automated analysis

Using DeepLabCut (Mathis et al., 2018), we assessed the effects of whisker trimming on the posture of the animals within the start zone of the Y-maze. Four markers (snout, left ear, right ear, tail base) were used to build a simple postural skeleton (Fig. 1D). We trained the deep neural network for 200,000 iterations on 500 labeled frames. This training regimen produced a good fit of the model to the training data (loss <0.005), such that predictions of movements by DeepLabCut resulted in accurate tracking of body parts. We defined positions within 1.3 cm (0.5 inch) of each stimulus panel as the contact zone. We then computed the fraction of snout and tail base positions within the contact zone relative to total positions tracked, and compared these values across whisker trimming conditions (one randomly chosen day for intact whiskers versus post-trimming, day 4 for the three trimming experiments). Only points with a high likelihood measure (>0.7) were included in this analysis.

### Data analysis and statistics

The sample size used in our study (*N*=6; three animals per experimental group) is typical for psychophysical investigations of sensory perception (Picciotto, 2020; Smith and Little, 2018; Rouder and Haaf, 2018), especially those conducted in species other than standard laboratory rodents – given the challenges and costs of the extensive behavioral training necessary to make these measurements, combined with limitations in availability of animals. We use highly practiced, experienced animals as our subjects, and all measurements have been made in animals that display a specific, criterion level of performance in a baseline condition, placing all subjects at the same operating point. These characteristics of the study design are known to lower within-observer and between-observer variability in many psychophysical tasks (Smith and Little, 2018).

All data analyses and statistics were performed in MATLAB version 9.4.0 (MathWorks, Natick, MA, USA) and R version 3.6.1 (https://www.r-project.org/). Texture discrimination performance across different sandpaper combinations was primarily assessed using the metric of accuracy (percentage of correct trials). Psychometric functions were plotted for individual animals across different sandpaper combinations. These were used to obtain average psychometric functions, and texture discrimination threshold values across animals in each experimental group and testing condition. Texture discrimination threshold was defined as the smallest difference in sandpaper grit size for which animals showed above chance performance. Hit rate was defined as the proportion of times the animal chose the right arm of the Y-maze when the S+ stimulus was on the right (Fig. 1B). The false alarm rate is the proportion of times the animal chose the right arm when the S+ stimulus was on the left. The percentage of correct responses was computed as the average of the hit rate and correct rejection rate:(1)
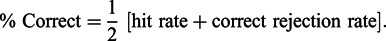


Signal detection theory (Green and Swets, 1966; Carandini and Churchland, 2013) was applied to calculate *d′* as a measure of discriminability; *d′* was calculated from the *Z*-score transform of the 2AFC percentage correct (National Research Council Committee on Vision, 1985) as follows:(2)
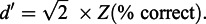
Response bias (*b*) was calculated using the equation:(3)
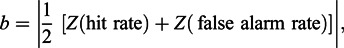
where *Z* is the inverse cumulative of the normal distribution.

All statistics reported were conducted using individual animals as the experimental units, following the guidelines for appropriately analysing datasets with subsamples (multiple observations per individual), as described in Lazic (2016). We deal with this in one of two ways: (1) by using summary measures after averaging across subsamples before statistical testing (each individual is a replicate, *N*=6 (3 SC, 3 EB) or (2) by including all subsamples without averaging, but specifying individual animal identity as a random factor in a linear mixed-effects model, followed by ANOVA.

Statistical significance of summary performance data relative to chance performance for individual animals was assessed using a two-tailed one sample *t*-test while comparisons of summary measures between groups were assessed using a two-tailed two sample *t*-test. Corrections for multiple comparisons were applied when necessary (Holm–Šidák correction). Mean texture discrimination functions were compared between different experimental groups (SC and EB) using a two-way ANOVA.

For analysis using linear mixed-effects models, outcomes of interest (Y, namely time data or performance accuracy), were modeled with experimental group (fixed factor with two levels, SC and EB) and test condition (time data: grouped by zone of the Y-maze; performance accuracy data: grouped by lighting condition/sandpaper condition/time relative to whisker trimming) as fixed factors, and individual animal identity (*N*=6) included as a random factor:(4)
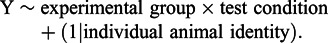
Tukey's *post hoc* test was used to make pairwise comparisons in linear mixed-effect model analyses.

## RESULTS

We trained sighted and early blind short-tailed opossums to perform a 2AFC texture discrimination task, in which choosing a rougher texture led to a food reward. By varying the difference in roughness between the textures presented for the choice, we generated psychometric functions for texture discrimination in short-tailed opossums and made comparisons of tactile perceptual sensitivity between sighted and early blind experimental groups.

### Sighted and early blind opossums successfully learned to base choices on texture cues to acquire a food reward

**Fig. 2.**
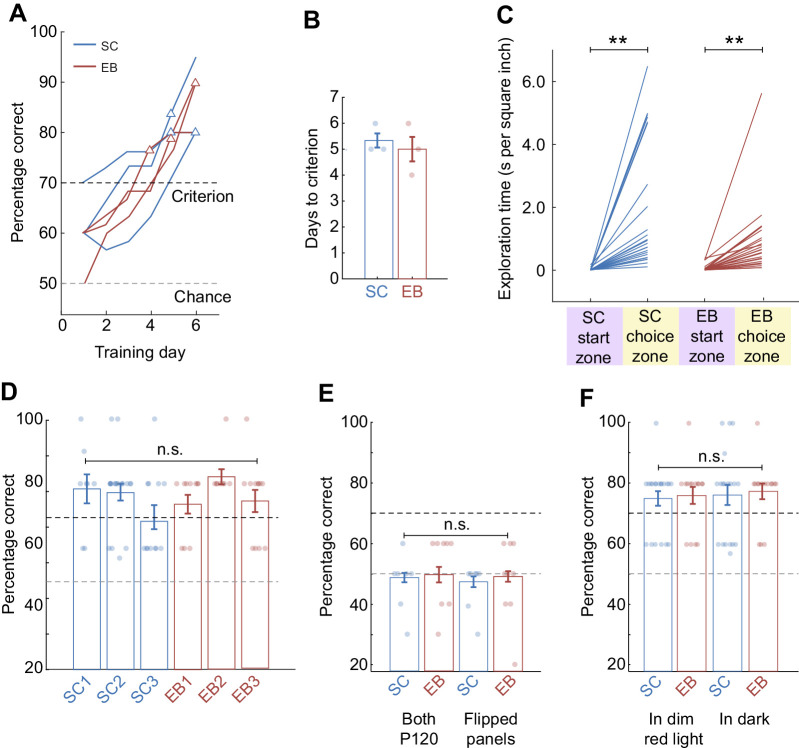
**Training results for short-tailed opossums performing the 2AFC texture discrimination task.** (A) Learning curves for four sighted control (SC, blue), and four early blind (EB, red) opossums, for discrimination of the S+ stimulus (P120 sandpaper) from the S– stimulus with maximal difference in texture (smooth panel). Learning curves show moving averages of performance over 3 days. Animals were considered to reach criterion (triangular markers) after two consecutive training days at ≥70%. (B) Both sighted and early blind opossums attained criterion in approximately five training sessions. Values are means±s.e.m. (C) Latency for texture discrimination during performance at criterion. On average, both SC and EB spent significantly more time in the decision zone versus the initial zone while performing the task, consistent with an active choice of the goal arm rather than selection by chance. Main effect of zone: *F*_1,4_=33.2, *P*=0.005, linear mixed-effects model ANOVA; group mean increase in choice zone versus start zone exploration time: SC, 1.89±0.40 s; EB, 0.80±0.19 s. **P<0.01. (D) After criterion was reached, P120 versus smooth discrimination performance was not significantly different between SC and EB groups. Values are means±s.e.m. Main effect of experimental group: *F*_1,4_=1.68, *P*=0.242, linear mixed-effects model ANOVA; group means: SC, 75.5±2.26%; EB, 77.2±2.19%. (E) Control for any possible olfactory cues from sandpaper. Presentation of the same texture on both sides (either P120 on both sides or the P120 versus P2000 combination with flipped panels, smooth side facing in) caused accuracy to drop to chance in both groups. Values are means±s.e.m. Main effect of experimental group: *F*_1,4_=0.226, *P*=0.660, main effect of sandpaper condition: *F*_1,4_=0.081, *P*=0.790, mixed-effects model ANOVA; group means, flipped panels: SC, 46.6±3.85%; EB, 51.3±4.12%; group means, both P120: SC, 45.7±2.75%; EB, 47.8±4.09%. (F) Control for any possible light cues due to the use of dim red light. Performance in the dark under infrared guidance was not significantly different from performance under dim red light for either group. Values are means±s.e.m. Main effect of experimental group: *F*_1,4_=0.238, *P*=0.651, main effect of lighting condition: *F*_1,4_=0.403, *P*=0.560, linear mixed-effects model ANOVA; group means, red light: SC, 75.2±2.36%; EB, 76.2±1.89%; group means, dark: SC, 75.5±2.34%; EB, 78.1±2.88%. n.s., not significant.

In initial training sessions, opossums were presented with only one possible stimulus combination, the S+ stimulus consisting of a P120 sandpaper panel, the choice of which was positively reinforced, and the single S− stimulus consisting of a smooth panel. This was done to benefit task learning by maximizing the textural contrast between S+ and S− stimuli. Animals from both experimental groups achieved criterion-level performance accuracy in choosing the S+ stimulus in ∼5 days (Fig. 2A,B; SC: 5.33±0.27 days; EB: 5.00±0.47 days) after training started (fifth stage of the gradual behavioral shaping process; see Materials and Methods). Once the task was learned, both groups of animals reliably spent much more time per unit area exploring in the choice zone of the Y-maze, compared with the start zone (Fig. 2C; all sessions with intact whiskers, start zone versus choice zone: main effect of zone, *F*_1,4_=33.2, *P*=0.005, linear mixed-effects model ANOVA; group mean increase in choice zone versus start zone exploration time: SC, 1.89±0.40 s; EB, 0.80±0.19 s) before entering into an arm of the maze and terminating the trial, indicating that the animals were engaged in the task, and making choices based on evidence accumulated from sensory cues in the maze. After criterion was attained, on average, performance remained stable for individual animals, and was similar across experimental groups (Fig. 2D; main effect of experimental group: *F*_1,4_=1.68, *P*=0.242, linear mixed-effects model ANOVA; group means: SC, 75.5±2.26%; EB, 77.2±2.19%). To confirm that animals were not guided by possible odorant cues associated with the sandpaper panel, we included a subset of trials where the P120 versus smooth panel combination was presented with the panels flipped such that texture cues associated with the S+ stimulus were eliminated (see Materials and Methods; Fig. 2E). This caused performance to drop to chance levels in all animals, and was not significantly different from performance when the same texture (P120) was presented on both sides (main effect of experimental group: *F*_1,4_=0.226, *P*=0.660; main effect of sandpaper condition: *F*_1,4_=0.081, *P*=0.790, mixed-effects model ANOVA; group means, flipped panels: SC, 46.6±3.85%; EB, 51.3±4.12%; group means, both P120: SC, 45.7±2.75%; EB, 47.8±4.09%). Additionally, we confirmed that no cues associated with the dim red light were guiding choices in sighted animals (Fig. 2F) by conducting a subset of testing sessions in the dark, under the guidance of only an infrared-enabled camera. Performance was not significantly different under dim red light and dark conditions (main effect of experimental group: *F*_1,4_=0.238, *P*=0.651; main effect of lighting condition: *F*_1,4_=0.403, *P*=0.560; linear mixed-effects model ANOVA; group means, red light: SC, 75.2±2.36%; EB, 76.2±1.89%; group means, dark: SC, 75.5±2.34%; EB, 78.1±2.88%). Thus tactile cues, and not odorant or light cues, were responsible for successful task performance in both sighted and blind animals.

### Facial whiskers made a primary contribution in guiding texture-based choices with differential effects of trimming in sighted versus early blind opossums

**Fig. 3.**
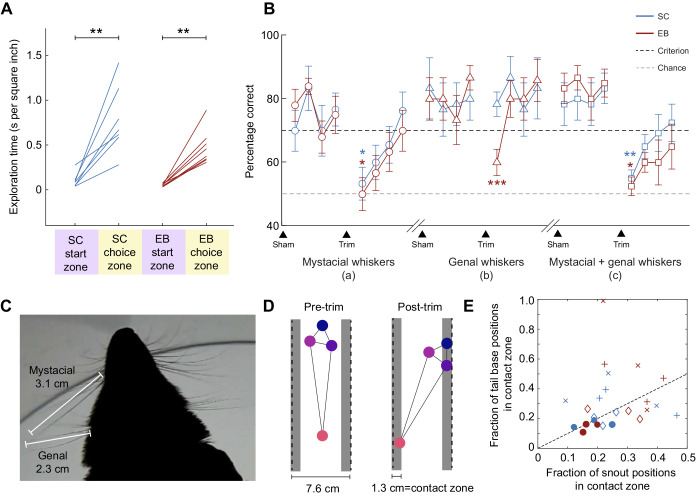
**Effects of whisker trimming on texture discrimination task performance.** (A) Latency for texture discrimination after trimming of all facial whiskers. Consistent with their initial training, sighted and early blind animals spent more time in the decision zone versus the initial zone even after whisker trimming. Main effect of zone: *F*_1,4_=50.2, *P*=0.002, linear mixed-effects model ANOVA; group mean increase in choice zone versus start zone exploration time: SC, 0.69±0.14 s; EB, 0.42±0.06 s. (B) Average texture discrimination accuracy across four consecutive testing sessions pre- and post-trimming of the facial whiskers: (a) mystacial whiskers (circles), (b) genal whiskers (triangles) and (c) mystacial and genal whiskers (squares). Whisker trimming acutely impaired performance on texture discrimination in both sighted and early blind opossums, which then gradually recovered over the next few sessions. Sham procedures in the pre-trimming phase did not induce a similar drop in performance. Mystacial whisker trimming (either alone or together with genal whisker trimming) diminished texture discrimination accuracy in both experimental groups, while genal whisker trimming affected only the early blind group. Values are means±s.e.m. Mystacial whiskers, main effect of time: *F*_1,4_=66.3, *P*=0.001, linear mixed-effects model with Tukey's *post hoc* test. Genal whiskers, main effect of time: *P*<0.001 linear mixed-effects model with Tukey's *post hoc* test. Mystacial+genal whiskers, main effect of time: *P*=0.002, linear mixed-effects model with Tukey's *post hoc* test. *P<0.05; **P<0.01; ***P<0.001. (C) Dorsal view of the opossum head showing mystacial and genal whiskers and the lengths of the longest whiskers in each subset for an average-sized opossum. (D) Example of a postural change observed in EB and SC opossums based on snout and tail base positions relative to the stimulus panels, after trimming of both mystacial and genal whiskers. (E) Fraction of snout and tail base positions within the contact zone of stimulus panels. Markers indicate individual animals (filled circles: intact whiskers; diamonds: genal whisker trimming; +, mystacial whisker trimming; ×, mystacial and genal whisker trimming). Animals with intact whiskers show relatively low fractions of direct snout and tail base contacts with stimulus panels, but this fraction becomes larger and more heterogeneous following whisker trimming.

We assessed the contribution of facial whiskers to texture discrimination in sighted and early blind short-tailed opossums by trimming the mystacial set of whiskers and the genal set of whiskers, either separately or in tandem. Following trimming of all facial whiskers, opossums continued to show similar patterns of exploration in the Y-maze as when whiskers were intact, but they spent significantly greater time per unit area in the choice zone compared with the start zone (Fig. 3A; post-whisker trimming, start zone versus choice zone: main effect of zone, *F*_1,4_=50.2, *P*=0.002, linear mixed-effects model ANOVA; group mean increase in choice zone versus start zone exploration time: SC, 0.69±0.14 s; EB, 0.42±0.06 s). For each experimental group, time spent within any particular zone was not significantly different between pre- and post-whisker trimming conditions (pre- versus post-whisker trimming group mean differences, start zone: SC, −0.06±0.04 s; EB, 0.04±0.03 s, *F*_1,4_=0.60, *P*=0.482; choice zone: SC, 0.97±0.88 s; EB, 0.45±0.20 s, *F*_1,4_=1.68, *P*=0.264, linear mixed-effects model ANOVA).

Thus, whisker trimming did not cause opossums to cease being engaged in task performance. Trimming of the mystacial whiskers led to an acute reduction in texture discrimination accuracy on the first day post-trimming and performance dropped to the level of chance in both experimental groups (Fig. 3B; SC: 53.3±5.17%, *P*=0.014; EB: 50±5.18%, *P*=0.017; main effect of time: *F*_1,4_=66.3, *P*=0.001, linear mixed-effects model with Tukey's *post hoc* test). This was followed by a steady recovery in discrimination accuracy back to criterion levels over the next 3 days, suggesting that opossums rapidly shifted strategies and relied on other body parts to perform texture discrimination, given that whisker regrowth would be minimal in 1–4 days post-trimming – although we cannot rule out the possibility that partial whisker regrowth may have contributed to performance recovery. Notably, trimming of the genal whiskers caused performance to significantly drop on the first day post-trimming only in early blind opossums (SC: 78.3±3.85%, *P*=0.724; EB: 60.0±4.15%, *P*<0.001; main effect of time: *P*<0.001 linear mixed-effects model with Tukey's *post hoc* test), and remained higher than chance even for the early blind group. By the second day following trimming of the genal whiskers, discrimination performance had fully recovered and stayed as such for the next two post-trim days.

Simultaneous trimming of both mystacial and genal whiskers in the third trimming experiment once again caused an acute deficit in whisker discrimination performance on the first day in both groups, similar to what was seen when mystacial whiskers alone were trimmed (SC: 55.0±2.65%, *P*=0.020; EB: 52.5±2.93%, *P*=0.020; main effect of time: *P*=0.002, linear mixed-effects model with Tukey's *post hoc* test). Importantly, sham procedures mimicking whisker trimming (see Materials and Methods) that occurred during the pre-trim phase for each trimming experiment did not cause a dip in performance for either group (Fig. 3B), indicating that poor performance on the first day after whisker trimming could not be attributed to non-sensory effects such as stress. Thus, when whisker input was present (especially from the mystacial whiskers), it was preferentially used to perform texture-based discriminations. Genal whiskers contributed to performance in early blind animals, but not in sighted animals. When whisker input was removed by trimming, performance recovered for both SC and EB opossums over the time course of a few days, possibly through an altered strategy that relied on tactile inputs from other body parts for texture discrimination.

We looked for postural differences in animals during whisker trimming experiments that would be indicative of the use of alternative tactile strategies. The start zone of the maze was designed to be 7.6 cm (3 inches) wide so that the longest macrovibrissae of opossums (Fig. 3C) would naturally brush against the stimulus panels during whisking, as animals ran through the maze. Thus, if animals are using a whisker-based strategy, the stimuli can be felt by the animal from close to the midline of the start zone. However, in the absence of whiskers, tactile strategies would require animals to move closer to the stimulus panels with the snout or torso in order to touch them (Fig. 3D). We examined the fraction of snout and tail base positions that fell within the contact zone relative to total tracked positions, to test for anterior and posterior postural changes. We found that in animals with intact whiskers, the fraction of snout and tail base contacts with the stimulus panels were both relatively low and tightly clustered across individuals (Fig. 3E; filled circles).

However, across all individuals, whisker trimming resulted in a greater number of both snout and tail base contacts with the walls of the Y-maze as indicated by a leftward shift in the data points (Fig. 3E). Posture also became much more heterogeneous, with some animals favoring posterior postural changes, while others only showed a shift in snout position contacts, indicating anterior postural changes. Genal whisker trimming caused smaller shifts in position overall, and mostly favored anterior posture changes. There were no systematic differences between SC and EB groups.

### Early blind opossums showed enhanced texture discrimination accuracy and sensitivity relative to sighted controls

**Fig. 4.**
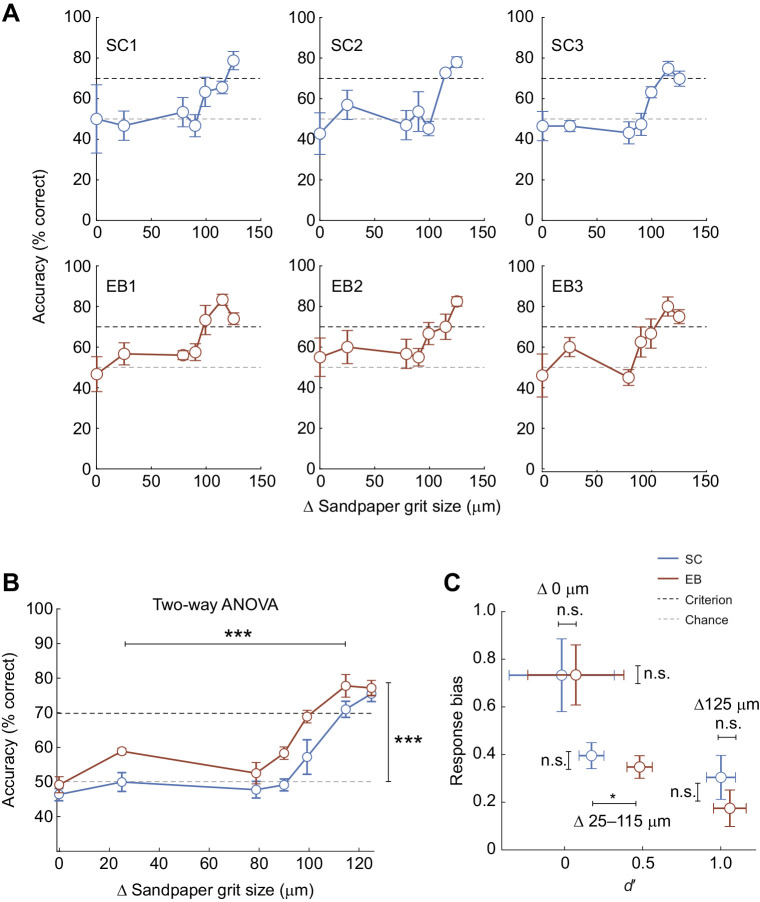
**Texture discrimination curves in sighted and early blind opossums.** (A) Psychometric functions for three individual sighted control opossums (top row) and three individual early blind opossums (bottom row). All animals have increased discrimination performance as differences in texture increased (Δ sandpaper grit size). Values are means±s.e.m. See Table 1 for full sample numbers per texture per animal. (B) Average psychometric functions for texture discrimination in early blind versus sighted control opossums. Discrimination accuracy increased with texture difference in both SC and EB animals, but the performance curves were shifted to the left for the EB group compared with the SC group, indicating lower texture discrimination thresholds in these animals. Early blind opossums could discriminate differences in texture of as small as 25 µm while sighted controls did no better than chance for <Δ100 µm differences. Values are means±s.e.m. Main effect of grit size difference: *F*_6,28_=26.8; *P*<0.001; main effect of experimental group: *F*_1,28_=15.1; *P*<0.001; two-way ANOVA. (C) Signal detection theory measures of texture discrimination performance. The scatterplot shows the relationship between mean *d′* and response bias measures for maximal (Δ125 μm), minimal (Δ0 μm) and intermediate (Δ25–115 μm) differences in texture. Only the sensitivity index, *d′*, was significantly different between SC and EB groups, for texture differences of 25–115 μm. Values are means±s.e.m.; 125 µm: SC, 0.73±0.15; EB, 0.73±0.13 SD units, *t*_4_=1.70, *P*=0.418; two sample *t*-test, Holm–Šidák correction for multiple comparisons; 0 µm: SC, 0.30±0.09; EB, 0.18±0.08, *t*_4_=0.032, *P*=0.976; two sample *t*-test, Holm–Šidák correction for multiple comparisons; 25–115 µm: SC, 0.40±0.05; EB, 0.35±0.05, *t*_4_=0.565, *P*=0.842; two sample *t*-test, Holm–Šidák correction for multiple comparisons. n.s., not significant. *P<0.05; ***P<0.001.

We varied the contrast between the textures of the S+ and S− stimuli in order to generate psychometric functions for texture discrimination in short-tailed opossums. Texture discrimination accuracy dropped with decreases in the difference between two presented textures, quantified as the difference in average grit size between sandpapers (Δ0–125 µm grit size). For individual animals in both SC and EB experimental groups, performance was at chance level for small differences in grit size and increased to criterion level for larger grit size differences (Fig. 4A). The average texture discrimination curve showed the same generally increasing relationship between textural contrast (Δsandpaper grit size) and discrimination accuracy for both groups. Importantly, there was a leftward shift in the EB curve relative to the SC curve (Fig. 4B). There was a significant main effect of grit size difference as well as experimental group on discrimination accuracy (grit size difference: *F*_6,28_=26.8; *P*<0.001; experimental group: *F*_1,28_=15.1; *P*<0.001; two-way ANOVA). On average, early blind opossums could discriminate differences in texture as low as 25 µm (P120 versus P150: EB group mean, 58.9±0.91%; *t*_2_=8.00, *P*=0.015, one sample *t*-test) while sighted controls did no better than chance for differences below Δ100 µm (P120 versus P600: SC group mean, 57.2±4.99%, *t*_2_=7.39, *P*=0.018, one sample *t*-test). Thus, early blind animals displayed an improved ability to discriminate smaller textural differences, compared with sighted controls under the same conditions.

We applied signal detection theory to separate the contributions of sensitivity (*d′*) and bias (*b*) to differences in behavioral performance between SC and EB animals (Fig. 4C). We computed *d′* and *b* when the difference in grit sizes was maximal (125 µm), minimal (0 µm) or in the intermediate range (25–115 µm). Improved texture discrimination accuracy in early blind animals for textural differences in the range of 25–115 µm was accompanied by significant improvements in sensitivity (*d′*) for those stimuli [group means, 25–115 µm: SC, 0.17±0.08 standard deviation (s.d.) units; EB, 0.48±0.08 SD units; *t*_4_=5.45, adjusted *P*=0.016; two sample *t*-test, Holm–Šidák correction for multiple comparisons], but not when the textural difference was maximal (group means, 125 µm: initially trained combination; P120 versus smooth: SC, −0.02±0.34 SD units; EB, 0.07±0.31 SD units; *t*_4_=0.352, adjusted *P*=0.934, two sample *t*-test, Holm–Šidák correction for multiple comparisons) or minimal (0 µm: P120 on both sides, or P120 versus P2000 flipped panels: SC, 1.00±0.09 SD units; EB, 1.06±0.11 SD units; *t*_4_=0.346, adjusted *P*=0.934, two sample *t*-test, Holm–Šidák correction for multiple comparisons). Response bias (*b*) was inversely related to grit size differences and sensitivity, being high for low Δgrit size stimulus combinations (where sensitivity was low) and low for high Δgrit size stimulus combinations (where sensitivity was high). Response bias did not differ significantly between experimental groups when grit size differences were maximal (group means, 125 µm: SC, 0.73±0.15; EB, 0.73±0.13 SD units, *t*_4_=1.70, *P*=0.418; two sample *t*-test, Holm–Šidák correction for multiple comparisons), minimal (group means, 0 µm: SC, 0.30±0.09; EB, 0.18±0.08, *t*_4_=0.032, *P*=0.976; two sample *t*-test, Holm–Šidák correction for multiple comparisons) or in the intermediate range (group means, 25–115 µm: SC, 0.40±0.05; EB, 0.35±0.05, *t*_4_=0.565, *P*=0.842; two sample *t*-test, Holm–Šidák correction for multiple comparisons). Thus, improved texture discrimination performance for smaller textural differences in early blind opossums can be attributed to improved perceptual sensitivity rather than differences in response bias between the two groups.

## DISCUSSION

### Texture discrimination in mammals

Textures consist of the microscale tactile features on the surfaces of objects, and provide important cues for sensory mediated behaviors in mammals (Diamond, 2010). Texture discrimination via the skin has been described for various tactile sensing arrays in mammals including the fingertips in humans (Lamb, 1983; Morley et al., 1983) and non-human primates (squirrel monkey, Hille et al., 2001; rhesus macaques, Connor et al., 1990; Connor and Johnson, 1992; Hollins and Bensmaia, 2007), the trunk in elephants (Dehnhardt et al., 1997), and the forepaws of rats (Bourgeon et al., 2004) and sea otters (Strobel et al., 2018). In the whisker system, texture discrimination has previously been described most extensively for rats and mice (Carvell and Simons, 1990; Arabzadeh et al., 2005; von Heimendahl et al., 2007; Wolfe et al., 2008; Diamond, 2010; Jadhav and Feldman, 2010; Pacchiarini et al., 2017), and in a few aquatic mammals, namely sea otters (Strobel et al., 2018), harbor seals (Dehnhardt et al., 1998), sea lions (Dehnhardt, 1994; Dehnhardt and Dücker, 1996) and manatees (Bachteler and Dehnhardt, 1999; Bauer et al., 2012). While the skin forms a continuous sheet that is densely innervated, the whisker array consists of a discontinuous grid, with touch receptors concentrated at discrete locations. Despite these differences in the peripheral morphology, it has been shown that texture discrimination via the whiskers can be just as sensitive as that mediated by the skin on human fingertips (Carvell and Simons, 1990; Bachteler and Dehnhardt, 1999; Diamond, 2010; Bauer et al., 2012; Strobel et al., 2018; O'Connor et al., 2020).

Short-tailed opossums have been shown to use surface texture as a cue to modify their locomotion (Lammers, 2009). Here, we found that sighted short-tailed opossums could discriminate between surfaces that differ in roughness by at least ∼100 µm (125 versus 25.8 µm mean grit size) while in the dark. Thus, short-tailed opossums are capable of using fine textural differences to guide behavior, as previously reported in rodents (Guic-Robles et al., 1989; Cybulska-Klosowicz and Kossut, 2001; Bourgeon et al., 2004; Aggestam and Cahusac, 2007). However, the smallest textural difference discriminated by sighted opossums in our study is considerably larger than that of rodents. Rats have been reported to discriminate between textures that differ by as low as ∼10–20 µm mean grit size (Morita et al., 2011). Mice can discriminate between novel and familiar textures separated by 25 µm in mean grit size (Wu et al., 2013; Wu and Dyck, 2018). It is important to note that the texture difference threshold measured is dependent on the roughness of the base stimulus used, as per Weber's law – when rats were tested relative to a rougher baseline sandpaper (P150, 100 µm mean grit size), the smallest differences in texture they could discriminate was up to 60 µm larger than when they were tested with a smooth (P1500, 12.6 µm mean grit size) baseline sandpaper (Morita et al., 2011). In our study, test comparisons were made with a relatively rough baseline texture (P120, mean grit size 125 µm); therefore opossums could be capable of discriminating smaller differences in texture than reported here, if a finer grit sandpaper is used as the baseline stimulus.

### Role of the facial whiskers in texture discrimination

Both sighted and blind opossums showed diminished performance on the texture discrimination task immediately following trimming of all facial whiskers. This could not be attributed to effects of anesthesia (Lipp and Van der Loos, 1991) or handling procedures associated with trimming because the same procedures minus whisker trimming did not yield a deficit in performance during the pre-trim phase for each set of trimming experiments. Thus, short-tailed opossums used facial whiskers for texture discrimination in this task.

However, trimming of subsets of facial whiskers led to differential effects in EB versus SC animals. Patterns of performance following mystacial versus genal whisker trimming indicated that SC animals used only mystacial whiskers for texture discrimination while EB animals used a strategy that integrated sensory information across both mystacial and genal whiskers. This suggests that even under normal circumstances mystacial whiskers are involved in fine texture discrimination but genal whiskers may perform different sensory functions than mystacial whiskers, as has been reported for other groups of whiskers in other mammals (for example, the whisker trident in rats; Thé et al., 2013; Chorev et al., 2016). In short-tailed opossums, both mystacial and genal whiskers are movable and are engaged in exploration of the environment through rhythmic back-and-forth motions during active whisking (Grant et al., 2013). The differential contribution of mystacial and genal whiskers to performance could be due to differences in active sampling strategies engaged during whisking and locomotion in blind versus sighted animals (as seen for the mystacial whiskers in blind rats and mice: Arkley et al., 2014; Grant et al., 2020) developed to compensate for the lack of vision. With the loss of a major sensory system (vision), genal whiskers appear to be recruited for making fine texture discriminations, indicating that the strategy employed for adaptive sensory mediated behaviors is highly flexible and dependent on available inputs from the different sensory systems.

There is some support for behavioral flexibility in sighted animals as well. In all trimming experiments for both experimental groups, texture discrimination performance recovered over the next 3 days when the trimmed whiskers had still not grown back. Given that we verified that olfactory and visual cues were not used to perform the discrimination task (Fig. 2E,F), it appears that both SC and EB animals utilized strategies mediated by other body parts for making tactile discriminations, namely increased snout and torso contacts with the textural stimuli (Fig. 3C–E). Recent work in our laboratory also demonstrated a similar finding in short-tailed opossums during a ladder rung walking task (Englund et al., 2020) – following whisker trimming, SC and EB animals held their snouts closer to the tactile substrate and exhibited significantly greater nose tapping behavior (physical contact of the snout with ladder rungs) while walking across a horizontal ladder with variable rung positions.

One possibility is that opossums adapted to performing texture discrimination task with partially regrown whiskers during the post-trim phase. Other possibilities include the use of microvibrissae (Kerekes et al., 2017; Kuruppath et al., 2014; Morita et al., 2011), skin on the snout (Kerekes et al., 2017; Morita et al., 2011) or possibly even skin or fur on the torso (Kerekes et al., 2017). Quick recovery of performance in the post-trim phase has also been reported in rats allowed to freely run while discriminating fine tactile patterns (Kerekes et al., 2017). Notably, once whiskers had fully grown back, we found that opossums returned to using a whisker-based strategy during the texture discrimination task – as was evident from the reduction in performance seen once again in the post-trim phase following the last trimming experiment, when both sets of facial whiskers were trimmed. In the absence of visual information either temporarily (in the case of SC animals) or over the course of development (in the case of EB animals), opossums favored a whisker-based strategy to discriminate between textures. Thus, while opossums were pre-disposed to using their whiskers for texture discrimination, they may flexibly recruit alternative strategies based on available sensory inputs, over the course of their lifetime. Such behavioral flexibility, which is probably cortically mediated, is a common feature in mammals (Krubitzer and Prescott, 2018).

### Neural mechanisms of whisker-mediated texture discrimination

While whisker-based and cutaneous texture discrimination can provide similar levels of tactile sensitivity (Diamond, 2010), the peripheral differences in the associated touch receptor organization do imply differences in the underlying neural mechanisms (for a review, see O'Connor et al., 2021). In primate fingertips, for example, the dense packing of touch receptors on the skin surface allows for neural coding of textures based on the spatial configuration of particles that make up a texture (Hollins and Bensmaia, 2007; Diamond, 2010). However, in the case of whiskers, the spacing between particles that create textures is much smaller than the spacing between the whiskers themselves, thus a spatial code cannot be used. While spatial integration across greater numbers of whiskers can provide a more reliable texture signal (Lottem and Azouz, 2008), this is not necessary for texture discrimination; in fact there is evidence that textures can be discriminated with high accuracy using just a single whisker (von Heimendahl et al., 2007), based on neural coding of whisker motion upon contact with surfaces of different roughness. Thus, smaller somatosensory receptive fields may not benefit texture discrimination capabilities. Instead, the neural coding of stimuli applied to different whiskers on the face is thought to subserve information gathering on a larger spatial scale, such as the shape and position of objects in the environment (Diamond, 2010; Celikel and Sakmann, 2007), rather than texture.

### Enhanced tactile behavioral sensitivity following vision loss

In the current study, we demonstrate that texture discrimination performance in short-tailed opossums can be altered by developmental history – for the same sandpaper combinations, early blind opossums discriminated differences in textures by as much as ∼75 µm lower than the smallest discrimination made by sighted controls (EB: 25 µm versus SC: 100 µm). Several studies have reported enhanced tactile perception in early blind human subjects (Walker and Moylan, 1994; Goldreich and Kanics, 2003; Goldreich and Kanics, 2003; Legge et al., 2008; Alary et al., 2009; Wong et al., 2011; Ricciardi et al., 2014), although this was not demonstrated in all studies (Heller, 1989; Alary et al., 2009; Gurtubay-Antolin and Rodríguez-Fornells, 2017). Studies comparing different touch-based tasks revealed enhancement in sensory performance for some tasks but not others (Alary et al., 2009; Gurtubay-Antolin and Rodríguez-Fornells, 2017). Furthermore, other studies have shown that even for tasks in which early blind subjects showed superior tactile performance, this was seen for some body parts, but not others (Wong et al., 2011). Specifically, tactile spatial acuity was greater for the preferred reading finger of Braille readers compared with a non-preferred finger or other body parts, and was correlated with the level of use. These findings support the contribution of use-dependent mechanisms to the development of heightened performance via the spared senses.

Thus, whether or not a specific aspect of tactile performance is enhanced in blind individuals could depend on the behavioral strategies that they used to engage with their environment (Arkley et al., 2014; Schinazi et al., 2016), among other factors. This can be especially impactful over the course of development when experience can have a major influence on plasticity in the nervous system. Given that even sighted opossums were found to use a primarily mystacial whisker-dependent strategy to solve the texture discrimination task in the dark, it follows that increased reliance on the whiskers to perform this function in the absence of vision from an early age could result in use-dependent plasticity. Our study shows that opossums that are blind from a very early stage in development are capable of enhanced discrimination of finely scaled textures using a primarily whisker-dependent strategy. These findings add to our previous results from recordings in primary somatosensory cortex of early blind short-tailed opossums which provided evidence of smaller receptive fields and enhanced neural discrimination on a larger spatial scale, for tactile stimuli applied to different whiskers on the face (Ramamurthy and Krubitzer, 2018). In that study we found that S1 neurons in EB animals were more selective in their responses to the deflection of individual whiskers, especially along the rostrocaudal axis of the snout, in alignment with the primary axis of natural whisker motion. Together, these studies provide support for enhancement of the representation of tactile information across multiple spatial scales in short-tailed opossums following the loss of vision early in development.
